# The leukemia inhibitory factor (LIF) and p21 mediate the TGFβ tumor suppressive effects in human cutaneous melanoma

**DOI:** 10.1186/s12885-015-1177-1

**Published:** 2015-03-29

**Authors:** Laure Humbert, Mostafa Ghozlan, Lucie Canaff, Jun Tian, Jean-Jacques Lebrun

**Affiliations:** 1 Division of Medical Oncology, Department of Medicine, McGill University Health Centre, Montreal, QC Canada; 2 Department of Medicine, Royal Victoria Hospital, Suite H7.66, 687 Pine Avenue West, H3A 1A1 Montreal, QC Canada

**Keywords:** TGFβ/Smad signaling, Tumor suppression, Cell cycle arrest, Apoptosis, p21, LIF, STAT3, Melanoma, Cell migration

## Abstract

**Background:**

Cutaneous melanoma is the most lethal skin cancer and its incidence in developed countries has dramatically increased over the past decades. Localized tumors are easily treated by surgery, but advanced melanomas lack efficient treatment and are associated with very poor outcomes. Thus, understanding the processes underlying melanoma development and progression is critical. The Transforming Growth Factor beta (TGFβ) acts as a potent tumor suppressor in human melanoma, by inhibiting cell growth and preventing cellular migration and invasion.

**Methods:**

In this study, we aimed at elucidating the molecular mechanisms underlying TGFβ-mediated tumor suppression. Human cutaneous melanoma cell lines, derived from different patients, were used to assess for cell cycle analysis, apoptosis/caspase activity and cell migration. Techniques involved immunoblotting, immunohistochemistry, real time PCR and luciferase reporter assays.

**Results:**

We found the leukemia inhibitory factor (LIF) to be strongly up-regulated by TGFβ in melanoma cells, defining LIF as a novel TGFβ downstream target gene in cutaneous melanoma. Interestingly, we also showed that TGFβ-mediated LIF expression is required for TGFβ-induced cell cycle arrest and caspase-mediated apoptosis, as well as for TGFβ-mediated inhibition of cell migration. Moreover, we found that TGFβ-mediated LIF expression leads to activation of transcription of the cell cycle inhibitor p21 in a STAT3-dependent manner, and further showed that p21 is required for TGFβ/LIF-mediated cell cycle arrest and TGFβ-induced gene activation of several pro-apoptotic genes.

**Conclusions:**

Together, our results define the LIF/p21 signaling cascade as a novel tumor suppressive-like pathway in melanoma, acting downstream of TGFβ to regulate cell cycle arrest and cell death, further highlight new potential therapeutic strategies for the treatment of cutaneous melanoma.

## Background

Skin cancer is the most common type of cancer worldwide, with an annual occurrence of almost 3 million cases. Cutaneous melanoma is one of the most aggressive and lethal human tumor, accounting for 75-80% of skin cancer-related deaths [1]. Melanoma incidence has dramatically increased over the past decades and it is now the most common cause of cancer deaths among young people between the age of 20-35 [2]. Melanomas have been classified into four clinical grades on the basis of their histology and prognosis. Grade IV melanomas are highly metastatic and refractory to conventional chemotherapeutic and biological reagents. Most patients have localized disease at the time of the diagnosis and are cured by surgical excision of the primary tumor, but melanomas can be highly malignant, and can metastasize to various organs including skin, lung, liver, brain and bone [2]. The fifteen-year survival for stage I melanoma is 85% whereas it is only 5% for stage IV melanoma. [1]. Melanoma display multifactorial etiology, yet its genetic and immunological background have not been elucidated. Thus, understanding the molecular and signaling mechanisms underlying melanoma formation and progression is a prerequisite for the development of more efficient treatments. At the molecular level, several signaling pathways have been implicated in the control of melanoma tumor formation, including the Ras-Raf-Mek-Erk cascade, which often exhibits activating mutations in cutaneous malignant melanoma [3]. Other signaling pathways potentially implicated are PI3K/AKT, Wnt, NF-κB, Jnk/c-Jun, JAK/STAT and TGFβ [4]. Contrary to frequent *BRAF* mutations which occur at a frequency of 50-80% [4], no genetic alterations of TGFβ signaling molecules have been identified in melanomas that could explain their resistance [5].

TGFβ signaling is initiated by the type II receptor (TβRII), a constitutively auto-phosphorylated serine/threonine kinase, which upon ligand binding recruits and transphosphorylates the type I receptor (TβRI), thereby activating its kinase activity [6]. Activated TβRI then phosphorylates mediators known as receptor-regulated Smads (R-Smads), Smad2 and 3, and allows subsequent heterotrimerization with a common partner, Smad4 [7,8]. The Smad heterotrimer translocates to the nucleus where it can bind DNA and regulate transcription, along with transcription factors, co-activators or co-repressors [6].

The role of TGFβ in cancer is complex and ranges from cell growth inhibition to regulation of cell migration and invasion [6,9,10]. In several types of cancer, such as breast cancer, TGFβ exerts a dual role: while it acts as a potent cell cycle inhibitor and a pro-apoptotic factor in normal and premalignant states, these tumor suppressive effects are lost in more advanced tumors and replaced by tumor promoting effects leading to metastasis [6,9-11]. In melanocytic systems, the role of TGFβ is different. While TGFβ acts as a potent tumor suppressor in normal melanocytes through the regulation of the plasminogen activation system, it also inhibits cell migration and cell invasion in melanoma of various stages [12,13]. Regarding cell growth inhibition, it has been reported that normal melanocytes in culture are sensitive to the growth-inhibitory effects of TGFβ, whereas melanoma cell lines demonstrate various degrees of resistance to these effects [14,15]. However, TGFβ is perfectly capable of inducing Smad signaling and Smad-dependent transcription in melanomas, suggesting that desensitization to the anti-proliferative activity of TGFβ is highly specific to cell cycle progression [12,16]. Also, several studies have shown an increased expression and secretion of the TGFβ isoforms in melanoma cell lines compared to normal melanocytes, suggesting that TGFβ signaling is still active in these cells [14,17-20]. While it seems that TGFβ acts as a potent tumor suppressor in melanocytic systems, the TGFβ tumor suppressive mechanisms have not been thoroughly investigated in melanoma [21].

Previous work from our lab showed that TGFβ inhibits human cutaneous melanoma cell migration and invasion through regulation of the plasminogen activator system [12]. We found by analysis of the transcriptome of two human melanoma cell lines, WM793B (Vertical growth phase melanoma, VPG, Stage I) and WM278 (VPG, Stage II), that one particular gene, the leukemia inhibitory factor (LIF), appeared to be strongly upregulated by TGFβ. Two previous studies have reported the induction of LIF mRNA and/or protein by TGFβ in Schwann cells [22] and glioblastoma [23] and shown this upregulation to be Smad-dependant by binding to a Smad binding element in LIF promoter. LIF is a member of the interleukin 6 (IL-6) family of cytokines, which includes IL-11, IL-27 and Oncostatin M (OSM) [24-26]. LIF signals through LIF receptor (LIF-R) which shares the gp130 subunit with other members of its class and which activates the JAK-STAT pathway [24-26]. LIF is expressed at the embryo stage and in many adult cell types and has been shown to be crucial for blastocyst implantation, maintenance of hematopoietic stem cells, differentiation, cell growth, inflammation, cachexia in animals, mammary gland involution after lactation, neurogenesis, and tissue regeneration [25,26]. Its role in cell growth is unclear as it was shown to both positively and negatively regulate proliferation [24-26], suggesting that these effects may be tissue-specific. Promotor studies and CHIP assays have shown that members of the LIF family such as Oncostatin M and IL-6 upregulate p21 expression through the Jak/STAT pathway, but this has not yet been investigated in melanoma cells [27,28].

TGFβ has been shown to regulate the expression of p21, to suppress the expression of genes important for cell cycle progression and to induce the expression of genes important for senescence [9,11]. However, the role of p21 in apoptosis is paradoxical and more investigations are needed [29-35]. Several studies have reported that p21 was detected in primary melanomas and metastatic lesions, while p21 levels were low or undetectable in melanocytic nevi. p21 might play an important role in melanoma progression, but the mechanisms are unknown.

In the present study, we aimed to understand the molecular mechanisms underlying TGFβ growth inhibition and apoptosis in human melanoma cells.

## Methods

### Reagents

Recombinant human TGFβ and LIF were purchased from Peprotech (Dollard des Ormeaux, Quebec, Canada). Tissue culture medium RPMI1640 was from Hyclone (Logan, UT, US). FBS, antibiotics (penicillin/streptomycin) and Lipofectamine 2000 were from Life Science (Grand Island, NY, USA). Antibodies against LIF, p15, p21, c-myc, p-Stat3 (Tyr705), Stat3 and β-tubulin were from Santa Cruz Biotechnologies (Santa Cruz, CA, USA). Scrambled, p21 and LIF siRNAs were from Sigma (Oakville, ON, Canada). D-luciferin and Lumi-light plus were from Roche Diagnostics (Laval, Qc, Canada). MMLV reverse transcriptase and random primers were from Life Science (Grand Island, NY, USA).

### Cell culture

Cutaneous melanoma cell lines WM793B and WM278 cell lines were isolated from the primary tumors of a 37-year-old male patient and a 62-year-old female patient and were kindly provided by Dr Louise Larose (McGill University, Montreal, Canada). The WM278 cell line harbors a V600E mutation in the *BRAF* gene, and a hemizygous deletion of *PTEN. NRas* and *CDK4* are wild type. WM793B cells are positive for a V600E *BRAF* mutation and carry a W274X mutation as well as a hemizygous deletion of *PTEN*. This cell line also has a mutation K22Q of *CDK4*. NRas is wildtype.

Cells were cultured at 37°C in RPMI1640 medium supplemented with 10% FBS and antibiotics under a humidified atmosphere of 5% CO_2_.

### Cell cycle analysis

Melanoma cells were plated in 24-well plates, serum starved overnight, and treated or not with TGF-β (200 pM) for 24 hours in a medium containing 2% FBS. Cells were washed in PBS and fixed in ethanol 70% for 2 hours. When ready for analysis, cells were resuspended in a solution containing 50 μg/ml propidium iodide, 50 μg/ml RNAse A and 0.1% Triton X-100. Cell cycle analysis was measured using an Accuri C6 flow cytometer (BD Biosciences, Mississauga, ON, Canada).

### Quantitative real time PCR

Total RNAs were extracted with Trizol (Life Science, Grand Island, NY, USA) according to the manufacturer’s instructions. One μg of RNA was reverse transcribed using M-MLV reverse transcriptase and random primers. Amplification of cDNA was performed by quantitative real time PCR (qPCR) (Bio-Rad iQ Sybr Green supermix, Mississauga, ON, Canada and RotorGene Corbett, San Francisco, CA, USA). Human GAPDH was used as a housekeeping gene. The qPCR conditions were: 3 minutes 95°C, then 40 cycles of 10 seconds at 94°C, 10 seconds at 60°C and 20 seconds at 72°C.

### Immunoblotting

Cells were lysed for 30 minutes at 4°C in RIPA buffer (50 mM Tris-HCL pH 7.4, 150 mM NaCl, 1% triton X-100, 1 mM EDTA, 1 mM EGTA, 1 mM DTT) supplemented with protease inhibitors (1 mM PMSF, 10 μg/ml leupeptin and aprotinin and 2 μg/ml of pepstatin A). For analysis of phosphorylation, 100 mM sodium fluoride, 10 mM sodium pyrophosphate and 100 mM sodium ortho-vanadate were added. For analysis of LIF expression, conditioned media were concentrated using Amicon® Ultra-15 Centrifugal Filter Unit with Ultracel-30 membrane (Billerica, MA, USA). Total lysates or concentrated media were immunoblotted by SDS-PAGE against specific antibodies. Immunoreactivity was revealed by chemiluminescence using Lumi-light PLUS (Roche, Mississauga, ON, Canada). Protein levels were quantified by densitometric analysis (ImageJ software, http://rsb.info.nih.gov/nih-image/).

### Apoptosis assay

Melanoma cells were plated in 96-well plates, starved overnight, then treated or not for 72 hours with TGFβ (200 pM) in medium supplemented with 2% FBS. Caspase 3/7 activity was measured by luminescence using Caspase 3/7 assay (Promega, Madison, WI, USA) according to the manufacturer’s instructions.

### Promoter-reporter constructs transfection and luciferase assay

The p21-luc and p21inr-luc constructs were kindly provided by Dr Xiao Fan Wang. For transient transfection, WM278 or WM793B cells were plated in 6-well dishes in RPMI1640, 10% FBS (1-4 × 10^5^ cells per well), and incubated overnight. The next day, cells were transfected with Lipofectamine reagent with 80nM siRNA. After 24 hours cells were transfected with 1 μg of promoter-reporter construct and 0.5 μg of Renilla luciferase construct per well. The following day, cells were serum-starved in RPMI overnight and cultured with or without 100 pmol TGF-β for 16 h. Cells were washed in PBS and lysed in 250 μl of passive lysis buffer (25 mM glycylglycine, 15 mM MgSO_4_, 4 mM EGTA, 1 mM DTT and 1% Triton X-100) on ice. Supernatants were collected by centrifugation (12,000 rpm, 20 minutes, 4°C). Luciferase activity was measured in a Fluostar Optima luminometer (BMG Labtech) using 45 μl of cell lysate and D-luciferin. Firefly luciferase activity was normalized to Renilla luciferase activity.

### Immunohistochemistry

Tissue sections (5 μm) from a melanoma microarray slide (ME1004a, US Biomax) were stained for p-Smad3 and LIF at the Goodman Cancer Research Histology core laboratory (McGill University, Montreal, Canada). LIF staining was revealed using Bajoran purple chromogen and p-Smad3 was developed using 3,3′-Diaminobenzidine (DAB) staining. The staining was scored from 0 to 4, where 0 means no staining while 4 means a strong staining. Representative pictures were taken at 40X magnification.

### Migration assay

Cells were plated on top of 24-well cell culture Transwell inserts (BD Biosciences, Mississauga, ON, Canada) and stimulated or not with TGFβ (200 pM) for 48 hours after an overnight serum starvation. The bottom chambers contained medium supplemented with 10% FBS as a chemoattractant. The migratory cells located on the filter of the bottom chamber were fixed for 10 minutes in paraformaldehyde and stained with 0.5% crystal violet. Images were taken using phase contrast light microscopy and migratory cells were counted using ImageJ software.

### Ethics and consent

This study did not require any ethics statement or any written informed consent for participation from participants, as no participant, patient tissue samples but only cell lines in culture were used in the study.

## Results

### TGFβ induces cell cycle arrest and apoptosis in human cutaneous melanoma cell lines

**Figure 1:**
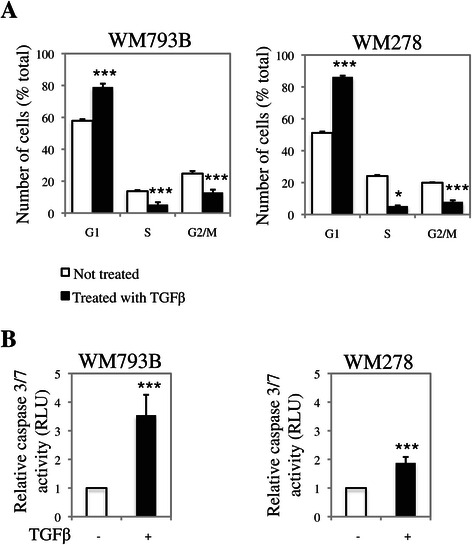
**TGFβ exerts strong growth inhibitory effects in various cutaneous melanoma cell lines. A**, WM793B and WM278 cells were treated or not with TGFβ and their cell cycle distribution was analyzed by propidium iodide staining. Data is graphed as the mean of the percentages of cells in each phase for at least 3 independent experiments. The error bars are the standard errors of the mean. For statistical analysis the *t*-test was performed compared to the non-treated control (***p < 0.001, *p < 0.05). **B**, WM793B and WM278 cells were treated or not with TGFβ and apoptosis was determined by measuring the caspase3/7 activity. Data is graphed as the geometric mean of relative luciferase units normalized to the non-treated control for at least 3 independent experiments. The error bars are the standard errors of the mean. For statistical analysis the z-test was performed compared to the non-treated control (***p < 0.001).

We previously found TGFβ to decrease cell viability in multiple melanoma cell lines, isolated from different patients [12]. To further investigate the mechanisms by which TGFβ regulates cell growth, we analyzed its effects on both cell cycle regulation and apoptosis. First, we analyzed the cell cycle profile of two cutaneous melanoma cell lines, WM793B and WM278, treated or not with TGFβ for 24 h. Following ethanol fixation of the cells and propidium iodide staining, the cell cycle profile was analyzed by flow cytometry. As shown in Figure 1A, WM793B and WM278 responded well to TGFβ showing a significant induction of G1 arrest. These results indicate that, while TGFβ growth inhibitory responses are lost in some melanomas, this growth factor still efficiently induces cell cycle arrest. We then analyzed the TGFβ effects on the regulation of apoptosis in these melanoma cell lines. Briefly, melanoma cells were treated or not with TGFβ for 72 h and caspase 3/7 activity was measured using a luminescent assay. Interestingly, as shown in Figure 1B, TGFβ significantly induced cell death, indicating that it acts as a pro-apoptotic factor in human melanoma and that its tumor suppressive effects are mediated through both cell cycle arrest in the G1 phase and caspase-mediated cell death.

### LIF upregulation by TGFβ is required for TGFβ-mediated cell cycle arrest and apoptosis

**Figure 2:**
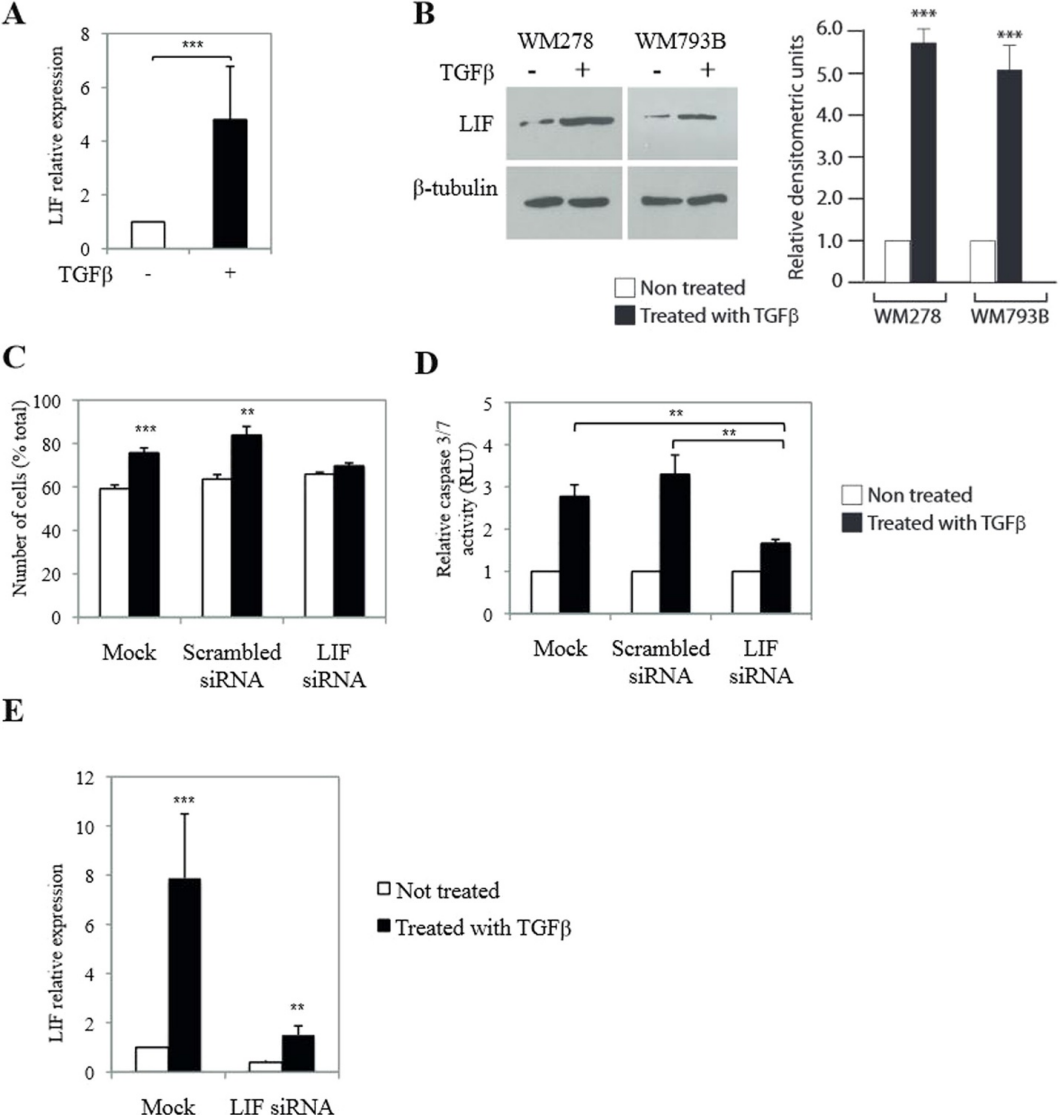
**TGFβ mediates its effects through LIF regulation. A**, WM278 cells were treated or not with TGFβfor 24 h and LIF expression was analyzed by qPCR. Error bars are standard deviations and z-test was performed (***p < 0.001). **B**, WM278 and WM793B cells were treated or not with TGFβ for 24 h and LIF expression was analyzed by Western blot (left panel) after concentration of conditioned media. β-tubulin was used as control. Right panel: Densitometric analysis of LIF protein levels. Error bars are standard errors of mean and t-test was performed compared to non-treated control (***p < 0.001). **C**, WM278 cells transfected with scrambled or LIF siRNA 48 h earlier were treated or not with TGFβ for 24 h and apoptosis was determined by caspase3/7 activity. Error bars are standard errors of mean and z-test was performed compared to non-treated control (**p < 0.01, ***p<0.001). **D**, WM278 cells transfected with scrambled or LIF siRNA 48 h earlier were treated or not with TGFβ for 24 h and apoptosis was determined by caspase3/7 activity. Data is graphed as the geometric mean of relative luciferase units normalized to non-treated control for at least 3 independent experiments. Error bars are the standard errors of mean and z-test was performed compared to non-treated control (**p < 0.01). **E**, WM278 cells transfected with scrambled or LIF siRNA 48 h earlier were treated or not with TGFβ for 24 h. LIF expression was analyzed by qPCR. Data is graphed as the mean of fold induction of gene expression in response to TGFβ for at least 3 independent experiments. Error bars are the standard errors of mean and t-test was performed compared to mock and scrambled siRNA treated conditions (**p < 0.01).

Transcriptome analysis of WM793B and WM278 cells revealed that one particular gene, the leukemia inhibitory factor (LIF), appeared to be strongly upregulated by TGFβ (data not shown). This was further confirmed and quantified at both mRNA and protein levels. As shown in Figure 2A and B, TGFβ potently induced LIF expression both at the mRNA and protein levels in WM793B and WM278 cells, suggesting a role for LIF in mediating the TGFβ effects in melanoma cells. Both cell lines showed a 5 to 6 fold increase in protein levels, as quantified by densitometry analysis. Our results highlight LIF as a TGFβ target gene in melanoma, suggesting that it may play a role downstream of TGFβ-mediated growth inhibition in melanoma. As shown in Figure 2C, while a control scrambled siRNA showed no effect, blocking LIF expression was able to almost completely block the TGFβ effect on the induction of G1 arrest, indicating that LIF plays a major role in the TGFβ-induced cell cycle arrest. We then tested the effect of silencing LIF gene expression on the induction of caspase-mediated apoptosis by TGFβ in WM278 cells. As shown in Figure 2D, blocking LIF expression almost completely inhibited the TGFβ-mediated induction of apoptosis, showing that LIF is required for TGFβ-mediated apoptosis. Efficiency of the LIF siRNA knockdown was assessed by qPCR (Figure 2E). These results highlight the involvement of LIF in cell cycle arrest and caspase-mediated cell death upon TGFβ stimulation.

### TGFβ exerts its tumor suppressive effects in melanoma through regulation of the cyclin-dependent kinase inhibitor p21

**Figure 3:**
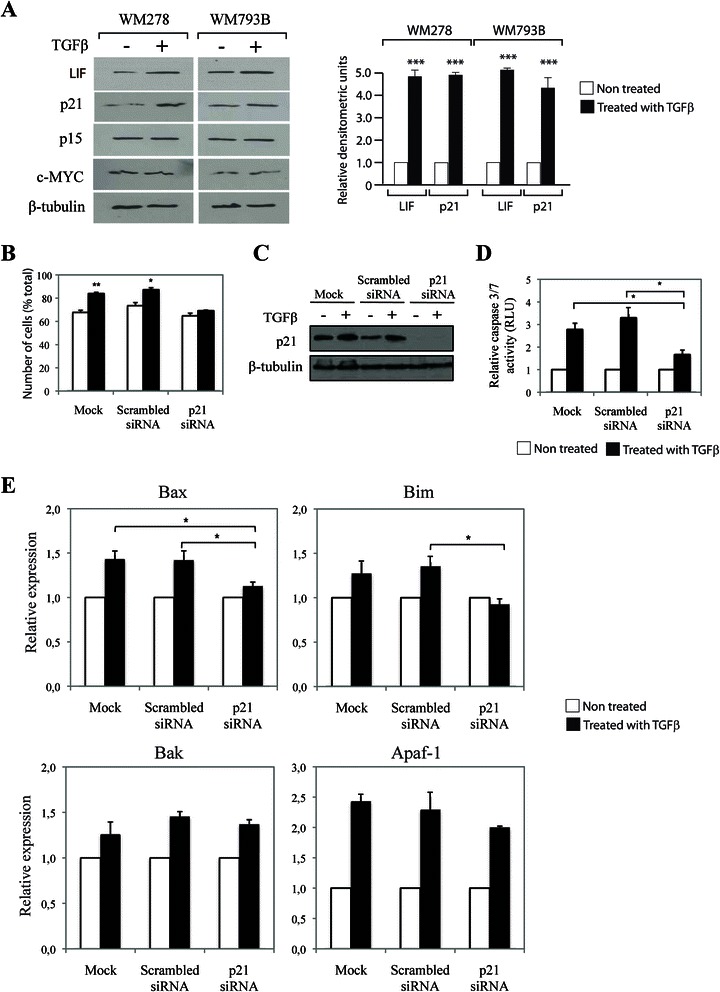
**TGFβ mediates its effects through p21 regulation. A**, WM278 and WM793B were treated or not with TGFβ for 24 h and expression of LIF, p21, p15, and c-MYC was analyzed by Western blot(left panel). β-tubulin was used as control. Right panel: Densitometry of LIF and p21 protein levels. Error bars are standard errors of mean and t-test was performed compared to non-treated control (***p<0.001). **B**, WM278 cells transfected with scrambled or p21 siRNA 48 h earlier were treated or not with TGFβ for 24 h and cell cycle distribution was analyzed by propidium iodide staining. Data is graphed as mean of percentages of cells in G1 phase for 3 independent experiments. Error bars are standard errors of mean and t-test was performed compared to non-treated control (**p < 0.01, *p < 0.05). **C**, WM278 cells transfected with scrambled or p21 siRNA 48 h earlier were treated or not with TGFβ for 24 h and p21 expression was analyzed by Western blot. β-tubulin was used as control. **D**, WM278 cells transfected with scrambled or p21 siRNA 48 h earlier were treated or not with TGFβ for 24 h and apoptosis was determined by caspase3/7 activity. Data is graphed as geometric mean of relative luciferase units normalized to non-treated control for at least 3 independent experiments. Error bars are the standard errors of mean and z-test was performed compared to non-treated control (*p < 0.05). **E**, WM278 cells transfected with scrambled or p21 siRNA 48 h earlier were treated or not with TGFβ for 24 h and gene expression was analyzed by qPCR. Data is graphed as mean of fold induction of gene expression in response to TGFβ for at least 3 replicates. Error bars are standard errors of mean and t-test was performed compared to mock and scrambled siRNA treated conditions (*p < 0.05).

To further understand how TGFβ/LIF regulate growth inhibition in melanomas, we examined the expression levels of several cell cycle regulators that have been shown to be involved downstream of TGFβ-mediated growth arrest in other tissues. These include p15 and p21 that were shown to be induced by TGFβ [36-38] and the oncogene c-MYC that was found to be downregulated by TGFβ in keratinocytes [39]. We examined the TGFβ effects on the expression levels of these downstream mediators in WM793B and WM278 cells. As observed by immunoblot analysis in Figure 3A, while there was no change in the protein expression levels of p15 or c-MYC, TGFβ significantly induced p21 protein, suggesting that p21 may act as the main cell cycle regulator downstream of TGFβ in human melanoma. To further address the role of p21 in these effects, we transfected WM278 cells with a specific p21 siRNA or a scrambled sequence as a negative control and examined the TGFβ effects on cell cycle arrest. As shown in Figure 3B, TGFβ significantly induced G1 arrest in the mock and scrambled siRNA conditions. However, when p21 expression was silenced, the TGFβ effect was completely abolished, suggesting that p21 not only is required downstream of TGFβ to mediate cell cycle arrest in melanoma cells but also plays a central role in the regulation of these events. The efficiency of the p21 siRNA was demonstrated by Western blot, using a specific p21 monoclonal antibody (Figure 3C). p21 has also been linked to the apoptotic process, however its exact function remains unclear and controversial, as it was shown to inhibit apoptosis in lymphoma cells [29], primary fibroblasts [30], and hepatoma cells [31], while it promotes apoptosis in ovarian cancer cells [32], hepatocytes [33] and hepatocarcinoma cells [34], and thymocytes [35]. Thus, we investigated whether TGFβ-mediated p21 expression in melanoma cells was required for the mediation of the TGFβ pro-apoptotic effects. As shown in Figure 3D, silencing p21 expression with a specific siRNA almost completely blocked TGFβ-mediated caspase-mediated cell death, defining a new role for p21 in melanoma as a pro-apoptotic factor. While the mechanisms through which p21 regulates the cell cycle have been relatively well documented, its effects on apoptosis are poorly understood. In order to elucidate how p21 promotes apoptosis downstream of TGFβ, we analyzed the expression of several pro-apoptotic genes known to be regulated by TGFβ (Bax, Bim, Bak and Apaf-1) [40]. As shown in Figure 3E, TGFβ induced the expression of all tested genes in melanoma cells. However, using a RNA interference approach, while we found TGFβ-mediated Bax and Bim gene expression to be p21-dependent, regulation of Bak and Apaf-1 by TGFβ does not involve p21. This indicates that p21 mediates some of the pro-apoptotic effects of TGFβ by inducing the expression of specific pro-apoptotic genes in melanoma. Together, our results highlight p21 as an important regulator of both TGFβ-mediated cell cycle arrest and apoptosis in human melanoma.

### LIF is required for TGFβ-mediated p21 upregulation

**Figure 4:**
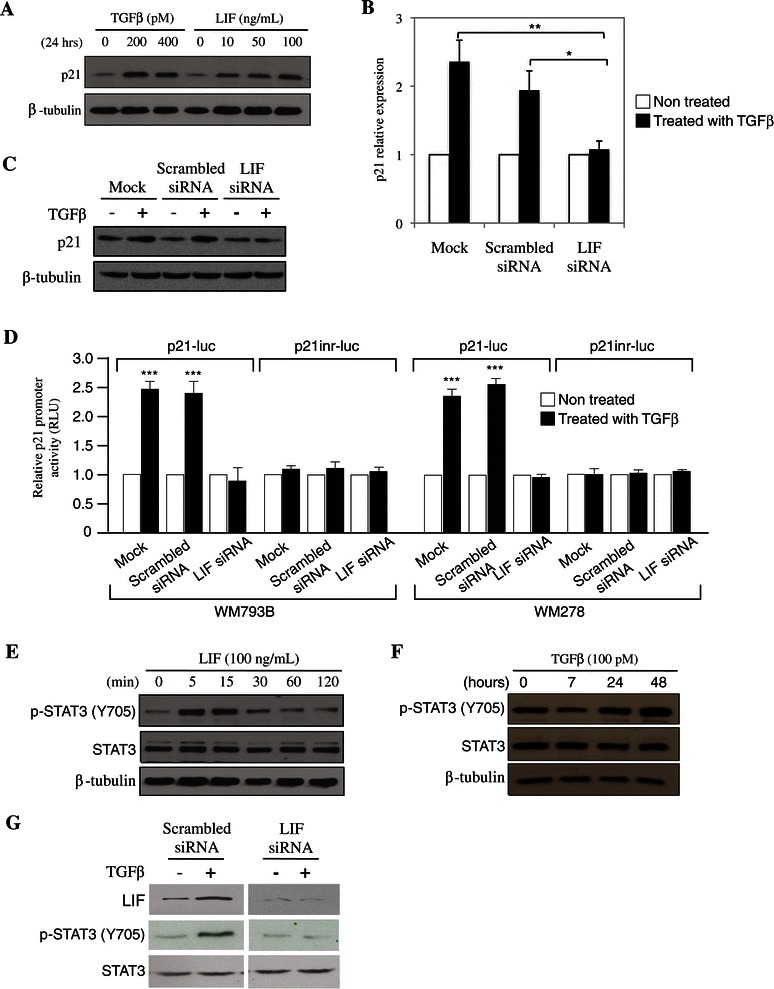
**TGFβ-mediated LIF upregulation regulates p21 expression at the transcriptional level. A**, WM278 cells were treated or not with TGFβ and LIF for 24 h and p21 expression was analyzed by Western blot. β-tubulin content was used as a control. **B**, WM278 cells transfected with a scrambled or a LIF siRNA 48 h earlier were treated or not with TGFβ for 24 h and p21 expression was analyzed by qPCR. Data is graphed as the mean of the fold induction of p21 gene expression in response to TGFβ for at least 3 biological replicates. The error bars are the standard errors of the mean. For statistical analysis the *t*-test was performed compared to the mock and scrambled siRNA treated conditions (*p < 0.05, **p<0.01). **C**, WM278 cells were transfected with a scrambled or a LIF siRNA 48 h earlier were treated or not with TGFβ for 24 h and p21 expression was analyzed by Western blot. β-tubulin content was used as a control. **D**, WM278 and WM793B cells transfected with a scrambled or a LIF siRNA 24 h earlier were transfected with the p21 luciferase reporter and the Renilla luciferase constructs, treated or not with TGFβ for 24 h, lysed and assessed for luciferase activity. Data is graphed as the arithmetic mean of relative luciferase units normalized to Renilla luciferase activity for 3 independent experiments. The error bars are the standard errors of the mean. For statistical analysis the *t*-test was performed compared to the non-treated control (***p<0.001). **E**, WM278 cells were treated with LIF (100 ng/mL) or **F**, with TGFβ for different period of times or **G**, with scrambled or LIF siRNA in the presence or absence of TGFβ, and phosphorylation of STAT3 was analyzed by Western blot. Total STAT3 content was used as a control.

Our results indicate that both p21 and LIF play an important regulatory role downstream of TGFβ in regulating melanoma growth inhibition. Interestingly, Oncostatin M, a member of the LIF family had previously been shown to induce p21 expression in osteoblastic cells [27,28]. This led us to investigate whether LIF could also regulate p21 gene expression, thereby linking LIF and p21 to TGFβ-mediated cell growth inhibition. We first assessed the link between LIF and p21 by treating WM278 cells with LIF and showed that LIF stimulation led to p21 upregulation comparable to TGFβ treatment (Figure 4A). To then investigate whether TGFβ-mediated p21 gene expression was LIF-dependent, we silenced LIF gene expression in WM278 cells and analyzed the effect of p21 regulation by TGFβ on both mRNA and protein levels. While p21 mRNA and protein expression were upregulated by TGFβ in the mock and scrambled siRNA conditions (Figure 4B and C), blocking LIF expression using a siRNA completely blocked this effect, indicating that LIF is required for TGFβ-mediated p21 upregulation. Furthermore, we found this effect to take place at the transcriptional level, as LIF gene expression knockdown using a specific LIF siRNA completely blocked TGFβ-induced p21 gene promoter activity. Indeed, as shown in Figure 4D, in WM278 and WM793 cells transfected with the p21-luciferase reporter construct (p21-luc) in the presence or the absence of a scrambled or LIF specific siRNA, the TGFβ-induced luciferase activity was completely blocked when LIF expression was knockdown. As LIF signaling is mediated through activation of the transcription factor STAT3, and as the p21-luc construct contains a STAT3 binding element [27], we next assessed whether TGFβ-induced p21 expression in melanoma was STAT3-dependent. For this, we used a second reporter construct (p21inr-luc) in which the STAT3 binding element has been removed [41]. Interestingly, TGFβ was unable to activate the p21 gene promoter in the absence of the STAT3 binding element (Figure 4D). These results indicate that LIF and its downstream effector STAT3 are required for TGFβ to induce p21 gene expression at the transcriptional level**.** STAT3 is the major effector of LIF and as shown in Figure 4E, stimulation of WM278 cells with LIF rapidly induces phosphorylation of STAT3. As we found TGFβ to increase LIF expression levels, we then investigated whether TGFβ could lead to STAT3 activation. As shown in Figure 4F, TGFβ stimulation of WM278 cells resulted in a significant increase in STAT3 phosphorylation, thus indicating that STAT3 is a downstream effector of the TFGβ pathway in these cells. Finally, to show that this increase in STAT3 activation by TGFβ was mediated through LIF, we treated WM278 and WM793B cells with LIF siRNA and treated or not with TGFβ. As shown in Figure 4G, knocking down LIF blocked the phosphorylation of STAT3 by TFGβ, further demonstrating that LIF is required for this regulation.

### LIF is required for the anti-metastatic effects of TGFβ in a p21-independent manner

**Figure 5:**
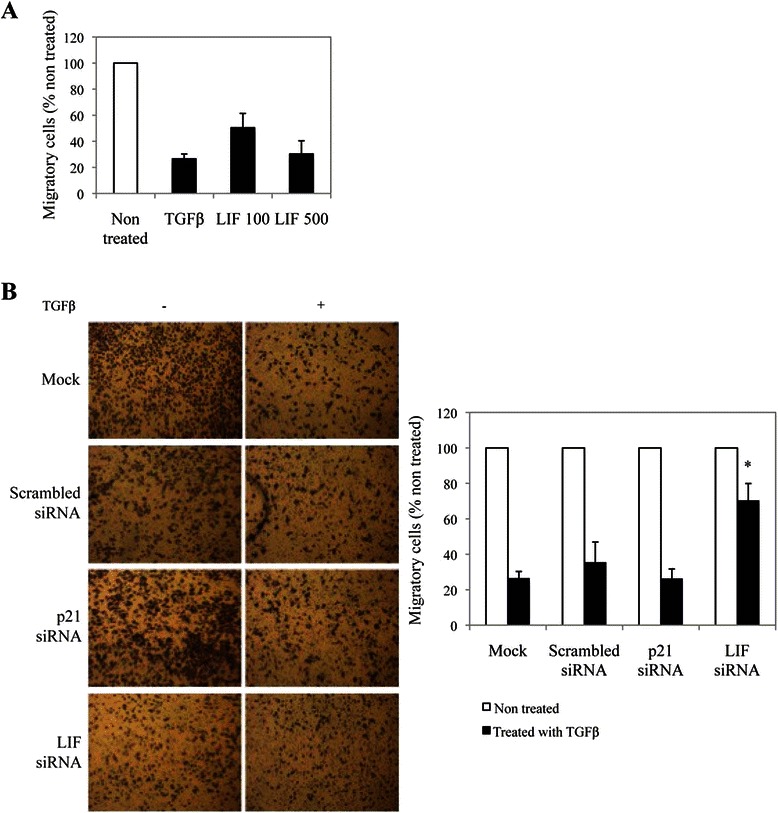
**LIF mediates the TGFβ-dependent inhibition of melanoma cell migration. A**, WM278 cells treated with LIF (100 or 500 ng/mL) or **B**, transfected with a scrambled or a LIF or a p21 siRNA 48 h earlier were plated in starvation medium on top of 24-well cell culture Transwell inserts, stimulated or not with TGFβ for 48 h, and the migratory cells were labeled with crystal violet, photographed using phase contrast light microscopy. A representative image is shown for each cell line (left panel). Migratory cells were counted using the ImageJ software and graphed (right panel). Data is graphed as the mean of at least 3 biological replicates. Error bars are the standard errors of the mean. For statistical analysis the *t*-test was performed compared to the non-treated mock condition (*p < 0.05).

We previously showed that TGFβ is not only a tumor suppressor in cutaneous melanoma but also acts as an anti-metastatic agent, inhibiting both the migratory and invasive properties of melanoma cells [12]. Recent literature showed that LIF-R is a metastasis suppressor in breast cancer [42]. We thus investigated whether LIF signaling might also play such a role in melanoma, by regulating the TGFβ-mediated inhibition of migration and invasion. We thus aimed at determining whether TGFβ-mediated LIF gene expression could also mediate the TGFβ anti-migratory/invasive activities. For this, we assessed cell migration using the Transwell assay, as previously described [12]. As shown in Figure 5A, TGFβ strongly inhibited cell migration in melanoma cells. Moreover, treatment with LIF mimicked this TGFβ effect, indicating that LIF, in addition to mediate the TGFβ-mediated growth inhibition, may also be involved in the TGFβ anti-metastatic effects. This was confirmed using a specific LIF siRNA: while a scrambled siRNA showed no effect, blocking LIF expression significantly inhibit the TGFβ effect on melanoma cell migration by about 60% (Figure 5B). We then assessed if p21 had a role in this process using a specific p21 siRNA. Interestingly, blocking p21 expression had no effect on migration, indicating that LIF mediates TGFβ anti-migratory activities independently of p21 (Figure 5B**)**. Overall these results demonstrate that LIF is not only involved in the tumor suppressive effects of TGFβ but also in its anti-metastatic activities, and that different pathways downstream of LIF are involved in these processes.

### Low response to TGFβ and low LIF expression correlate with melanoma aggressiveness

**Figure 6:**
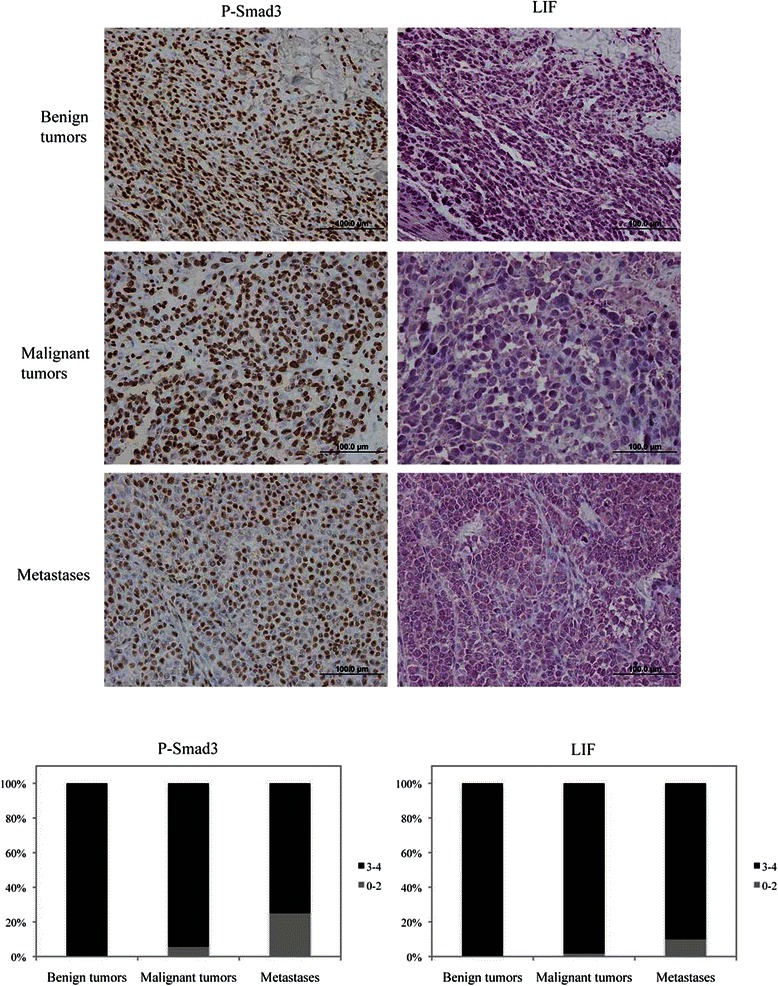
**The TGFβ/LIF pathway is lost across melanoma progression.** Tissue sections from a melanoma microarray slide were stained for P-Smad3 and LIF levels. The staining was scored from 0 to 4: (0, no staining of neoplastic cells, 1, weak staining, 2, weak to moderate staining, 3, moderate to strong staining, and 4, strong staining). Representative pictures were taken at the magnification 40X (upper panel). Data is graphed as the percentage of samples with levels 0-2 or 3-4 for each category (lower panel).

Having highlighted the TGFβ/LIF pathway as a potent tumor suppressor in melanoma, we then assessed the clinical relevance of these findings. For this, we analyzed phospho-Smad3 and LIF expression levels by immunohistochemistry in a tissue microarray consisting of 24 benign tumors, 56 malignant tumors, and 20 metastatic melanomas. Phospho-Smad3 was measured as an indicator of TGFβ signaling activity in these tumors. As shown in Figure 6, neoplastic cells in benign tumors showed high levels of phosphorylated Smad3, indicative of high TGFβ signaling activities. However, phospho-Smad3 levels were reduced in malignant tumors and even further diminished in the metastatic tissue samples. This is consistent with previous findings showing that normal melanocytes in culture are sensitive to the growth inhibitory effects of TGFβ, whereas melanoma cell lines demonstrate various degrees of resistance to TGFβ inhibitory effects, proportional to the tumor progression stage [14,15]. Interestingly, LIF expression levels exhibited a similar pattern, showing very high levels of expression in benign tumors, with a progressive decrease in malignant and metastatic tumors. These results indicate that while the TGFβ/LIF signaling axis acts as tumor suppressor pathway in the early stages of melanoma progression, it is partially disrupted in advanced stages, further emphasizing its essential role in the prevention of melanoma development and progression.

## Discussion

**Figure 7:**
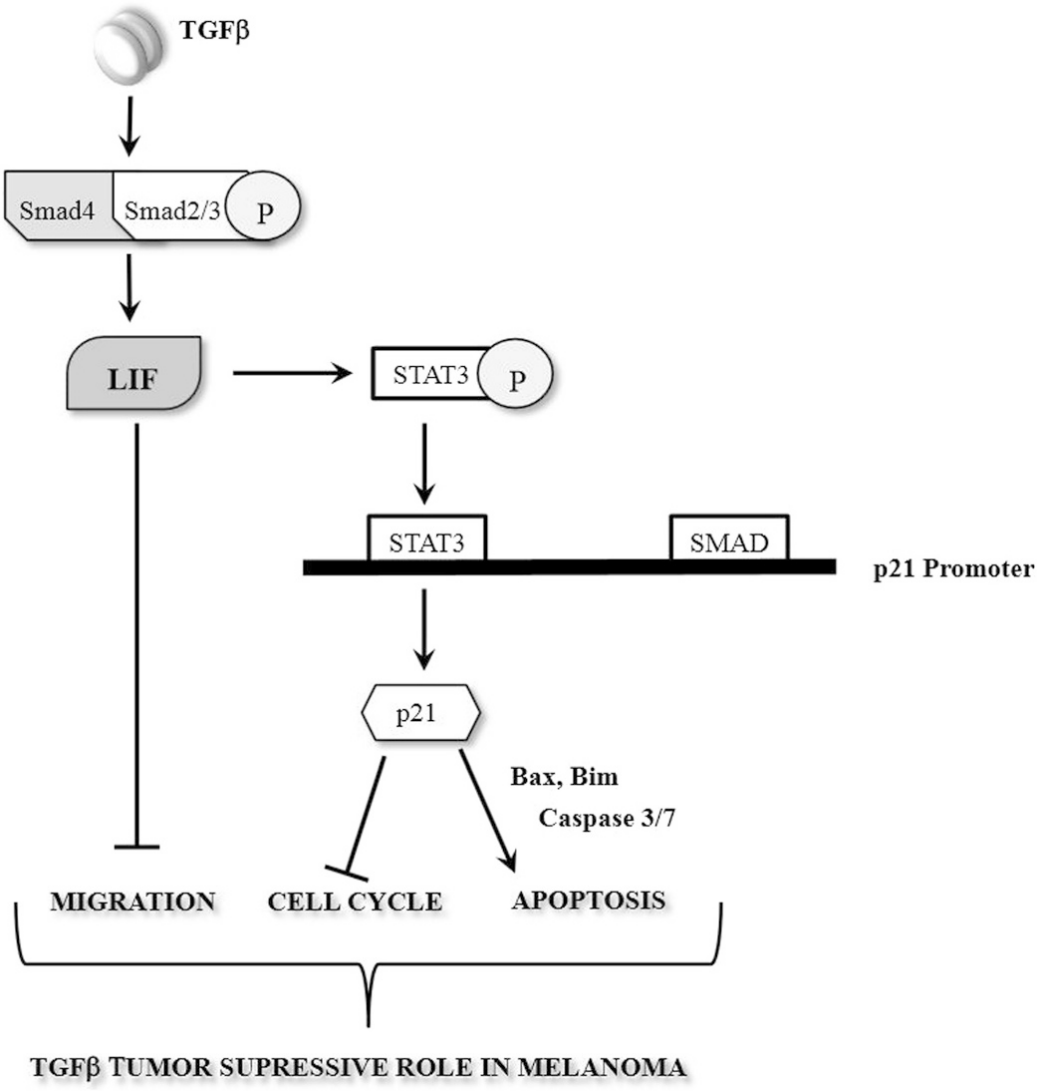
**Schematic showing the TGFβ/LIF- mediated tumor suppressive role in melanoma.** Our results show that TGFβ activates its canonical Smad signaling pathway, which in turn induces LIF secretion. LIF, via phosphorylating STAT3, triggers LIF binding to the p21 promoter, which consequently induces p21 gene expression, which elicits its inhibitory effect on cell cycle progression. Moreover, p21 induces apoptosis in a Caspase3/7 dependent manner. On the other hand, TGFβ-mediated activation of LIF inhibits migratory behavior in melanoma cell lines. Taken together, these results show the TGFβ/LIF-mediated tumor suppressive role in melanoma.

As illustrated in Figure 7, in this study, we showed that TGFβ regulates cell growth in melanoma not only by acting as a cell cycle inhibitor but also as a potent inducer of caspase-mediated cell death. We further dissected the intracellular mechanisms underlying these effects and found that the leukemia inhibitory factor (LIF) plays a critical role in mediating these tumor suppressive effects. Our results define LIF as a novel target downstream of TGFβ in melanoma cell lines and indicate that TGFβ-induced expression of LIF is a prerequisite for the TGFβ tumor suppressive effects, including cell cycle arrest and apoptosis as well as the inhibition of cell migration. In addition, we found that the cyclin-dependent kinase inhibitor p21 plays a significant role in mediating both G1 arrest and apoptosis, but not cell migration downstream of TGFβ. Moreover, we found that TGFβ-mediated p21 gene expression can induce expression of pro-apoptotic genes, such as Bax and Bim, leading to cell death. Our study defines a novel regulatory pathway mediated by the TGFβ/LIF/p21 signaling axis that controls tumor formation and tumor progression in melanoma.

The role of p21 as a cell cycle inhibitor has been well characterized in other tissues and cell types [37,43,44]. Through its amino-terminal CDK-cyclin inhibitory domain, p21 binds to both the cyclin subunit and the CDK subunit of CDK-cyclin complexes, preventing them from binding to p107, p130, and Rb, which are involved in cell cycle progression. p21 also directly inhibits DNA synthesis by disrupting DNA-polymerase binding to DNA. Nonetheless, the exact function of p21 in regulating apoptosis remains unclear and even controversial, as both pro- and anti-apoptotic p21 activities have been previously reported [45,46]. Many studies are indicative of an anti-apoptotic role for p21 in different target tissues. For example, p21-deficient lymphomas with a p53 deficient background showed a higher apoptotic rate than p21-proficient lymphomas, indicating a protective role for p21 against apoptosis [29]. Similarly, p21 depletion in human embryonic fibroblasts was reported to induce cell death in these cells [30]. In hepatoma cells, p21 was found to bind caspase 3, thereby preventing caspase activation and Fas-induced apoptosis [31]. In addition, p21 was also found to inhibit stress-induced apoptosis [47]. Indeed, p21 prevents stress-induced apoptosis mediated by the JNK and p38 signaling pathways by binding to and inhibiting the activity of the MAP3K5 (ASK1; MEKK5) in human rhabdomyosarcoma cells [48] and by binding to JNK kinases, further preventing their activation by upstream kinases [49]. On the other hand, multiple reports have also documented a pro-apoptotic role for p21. For instance, p21 overexpression in ovarian cancer cells was found to enhance susceptibility to cisplatin-induced apoptosis [32]. Reports showed that p21 could also facilitate deoxycholic acid-induced apoptosis in primary mouse hepatocytes [33] and ceramide-induced apoptosis in human hepatoma cells [34]. Similarly, thymocytes from mice carrying a p21 transgene targeted for restricted expression in the T cell lineage were found to be hypersensitive to radiation-induced programmed cell death [50]. These studies suggest that p21 can also act as a cell death inducer even though the molecular mechanisms underlying these effects are not fully elucidated. Altogether, these studies highlight the fact that the role of p21 in regulating cell death is clearly context-dependent. Our study indicates that, in the context of human cutaneous melanoma, p21 acts as a potent pro-apoptotic factor. We also showed that p21 acts downstream of the TGFβ/LIF signaling cascade and that it promotes caspase-dependent cell death though induced-expression of pro-apoptotic molecules, such as Bax and Bim.

Similarly, the role of LIF in cell growth regulation has not been clearly established. Evidence shows that LIF inhibits the differentiation of embryonic stem cells to maintain their pluripotentiality [51] and positively regulates the proliferation of germ cells, hematopoietic progenitors, megakaryocytes, myoblasts, and neural cells [52]. Conversely, LIF inhibits proliferation and induces differentiation of leukemic myeloid cells [53], promotes differentiation of adipocytes [54], cardiac muscle cells [55], and cardiac stem cells [56]. Exogenous LIF was reported to act as a growth factor for melanoblasts and melanocytes [57], yet another study showed that LIF did not exert any growth stimulatory effect in melanoma cells [58]. Our results clearly indicate that LIF inhibits melanoma cell growth downstream of the TGFβ signaling pathway. We found that LIF mediates both TGFβ-induced G1 arrest and apoptosis in melanoma, indicating a tumor suppressor-like role for this cytokine. This is also consistent with previous reports indicating that LIF could induce G1 arrest in medullary cancer cells [59] and retinal microvascular endothelial cells [60]. In terms of cell death, LIF has been reported to induce apoptosis in mammary epithelial cells [61], but was found to inhibit apoptosis in other cell types, such as olfactory sensory neurons [62] and myoblast cells [63,64]. Thus, similar to p21, LIF function as a regulator of apoptosis appears to be cell type- and tissue-specific.

Mounting evidence show that increased nuclear pSTAT3 expression in various solid tumors, such as lung [65], breast [66], head and neck [67], as well as thyroid [68,69], is correlated with either reduced tumor size, reduced aggressiveness or enhanced survival outcomes thus pointing towards a rather tumor-suppressive role of pSTAT3 in these cancers. Our results support these findings and show that that TGFβ-induced LIF expression through activation of STAT3 further leads to p21 gene transcription and TGFβ-mediated cell cycle arrest and apoptosis in melanoma.

Moreover, our study shows that in addition to its role in mediating TGFβ tumor suppressor effects, LIF also acts downstream of TGFβ to prevent tumor progression by inhibiting cell migration, in a p21-independent manner. This indicates that LIF is a major regulator of the TGFβ effects in cutaneous melanoma, not only relaying TGFβ-mediated cell cycle arrest and apoptosis but also TGFβ-mediated cell migration inhibition. Thus, our results define LIF signaling as a potent tumor suppressor and as a potential suppressor of metastasis in human melanoma. In fact, this is consistent with a recent study showing that LIF receptor (LIF-R) also acts as a metastasis suppressor in breast cancer [42]. In that context, LIF-R acts downstream of the microRNA miR-9 but upstream of Hippo signaling [42]. The authors further found that loss of LIF-R expression in non-metastatic breast cancer cells induced a metastatic behavior. Consistently, we show here that LIF itself also contributes to prevention of tumor metastasis in melanoma, by mediating the TGFβ inhibitory effects on cell migration.

## Conclusion

Collectively, our results indicate that the TGFβ/LIF/p21 signaling axis plays a major role in controlling tumor formation and tumor progression in melanoma. Interestingly, a recent clinical study from Tas et al., examining 60 patients with a pathologically confirmed diagnosis of melanoma, revealed that chemotherapy-responsive melanoma patients have higher levels of serum TGFβ compared to chemotherapy-refractory patients [70]. Moreover, melanoma patients with high levels of serum TGFβ also showed favorable overall survival compared to patients with lower levels [70]. These findings are in agreement with our results showing that TGFβ, via LIF/STAT3 activation, leads to the suppression of the invasive phenotype in melanoma and highlight TGFβ as a favorable prognosis marker and protective growth factor against tumor metastasis in human melanoma.
